# Cytotoxic Effect of Clerosterol Isolated from *Codium fragile* on A2058 Human Melanoma Cells 

**DOI:** 10.3390/md11020418

**Published:** 2013-02-06

**Authors:** Areum Daseul Kim, Youngki Lee, Sang-Hyuck Kang, Gi Young Kim, Hye Sun Kim, Jin Won Hyun

**Affiliations:** 1 School of Medicine, Jeju National University, Jeju 690-756, Korea; E-Mails: candy4860@nate.com (A.D.K.); yklee38@jejunu.ac.kr (Y.L.); 2 Department of Marine Life Sciences, Jeju National University, Jeju 690-756, Korea; E-Mails: kksghl0932@naver.com (S.-H.K.); immunkim@jejunu.ac.kr (G.Y.K.); 3 Cancer Research Institute, Seoul National University College of Medicine, Seoul 110-799, Korea; E-Mail: hyisun@snu.ac.kr

**Keywords:** clerosterol, melanoma cell, apoptosis, mitochondrial membrane potential

## Abstract

The cytotoxic effects and mechanism of action of clerosterol, isolated from the marine alga *Codium fragile*, were investigated in A2058 human melanoma cells. Clerosterol inhibited the growth of A2058 cells with an IC_50_ of 150 µM and induced apoptotic cell death, as evidenced by DNA fragmentation, an increase in the number of sub-G_1_ hypodiploid cells and the presence of apoptotic bodies. Clerosterol treatment caused the loss of mitochondrial membrane potential. Alterations in the expression of apoptosis-associated proteins in response to clerosterol treatment included upregulation of Bax, downregulation of Bcl-2 and activation of caspases 3 and 9. The pan-caspase inhibitor treatment attenuated the expression of the active form of caspases and cell death induced by clerosterol. The present results show that clerosterol exerts its cytotoxic effect in A2058 human melanoma cells by caspases-dependent apoptosis.

## 1. Introduction

Melanoma is the most aggressive form of skin cancer and is characterized by a high metastatic potential and extraordinary resistance to cytotoxic agents [1]. Despite recent advances, the results of chemotherapy in patients with metastatic melanoma remain inadequate due to the relative drug resistance of metastatic cells [2]. Apoptosis is mediated by two main pathways, the death receptor pathway (extrinsic) and the mitochondrial pathway (intrinsic). Most anticancer agents exert their cytotoxic effects by inducing apoptosis in tumor cells [3,4]. Therefore, apoptosis induction is vital for successful cancer treatment, and several chemotherapeutic drugs are known to induce apoptosis *in vitro* [5]. Seaweeds, which have anticancer properties, can be divided into three basic types: brown (Phaeophyta), red (Rhodophyta) and green (Chlorophyta). *Codium fragile* is a siphonous marine green alga belonging to the family Codiaceae. Marine algae produce a wide variety of natural compounds, which are usually referred to as secondary metabolites, because they are not involved in the basic machinery of life [6]. The importance of marine algae as a source of novel bioactive substances has been demonstrated recently, and several studies have identified and isolated biologically active compounds from algae. Furthermore, compounds isolated from marine algae have anticancer activity [7,8].

A recent report shows that the pro-apoptotic effects of siphonaxanthin (a marine carotenoid from green algae) in human leukemia cells are associated with the upregulation of GADD45α and DR5 (TRAIL receptor-2) expression and the downregulation Bcl-2 expression [9]. A glycoprotein extracted from the green alga *Capsosiphon fulvescens* stimulated pro-apoptotic signaling and inhibited the growth of human gastric cancer cells by inducing cell cycle arrest at the sub-G_1_ phase, which was associated with the downregulation of cyclin D, cyclin E, Cdk2, Cdk4 and Cdk6 and the upregulation of p21 and p27 [10]. A recent study showed that phycocyanin, one of the main biliproteins of blue-green algae, induced the generation of reactive oxygen species in tumor cells, which in turn induced apoptosis. Interestingly, phycocyanin downregulated the expression of Bcl-2, which is known to play an important role in the apoptotic death process [11]. The marine green algal genus *Codium* is an important source of clerosterol [12,13], whose molecule is a cholesterol derivative and is cytotoxic to A549 lung cancer cells [14]. The present study examined the mechanisms underlying the induction of apoptosis in A2058 human melanoma cells by clerosterol isolated from *Codium fragile*.

## 2. Results

### 2.1. Clerosterol Inhibits the Growth of A2058 Human Melanoma Cells

**Figure 1:**
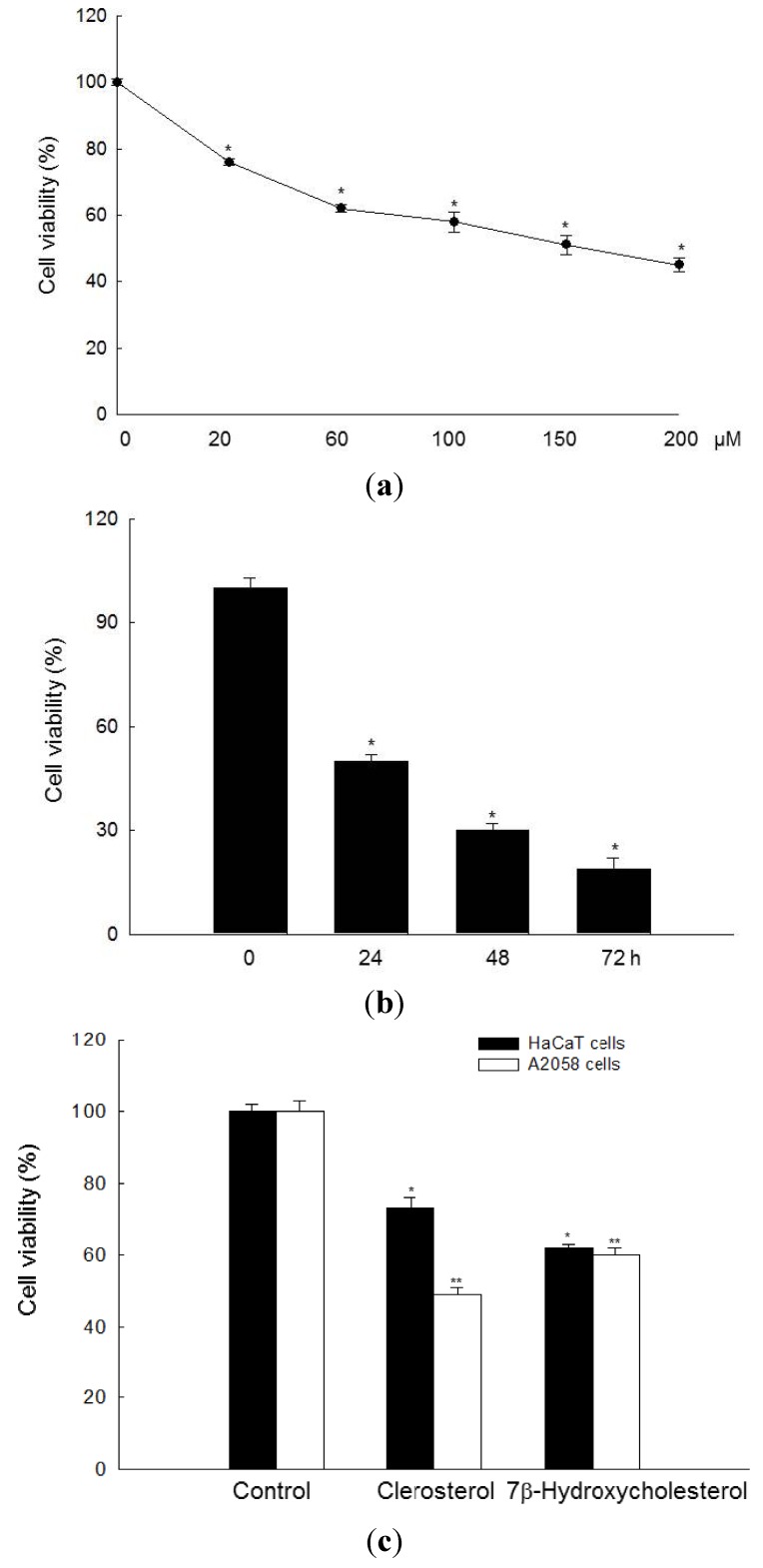
Cytotoxic effects of clerosterol in A2058 human melanoma cells and HaCaT human keratinocyte. Cell viability (**a**) was assessed using the MTT assay after treatment of A2058 cells with the indicated concentrations of clerosterol (20, 60, 100, 150, 200 µM) for 24 h and (**b**) after treatment with 150 µM clerosterol for the indicated times. * Significantly different from control cells (*p* < 0.05). (**c**) Cell viability was assessed using the MTT assay after treatment of HaCaT and A2058 cells with clerosterol and 7β-hydroxycholesterol at 150 µM for 24 h. *^, ^** Significantly different from control cells in HaCaT human keratinocyte and A2058 human melanoma cells (*p* < 0.05).

Clerosterol, whose structure belongs to a cholesterol derivative, significantly inhibited cell growth in a concentration-dependent manner. As shown in Figure 1a, the concentration of clerosterol required for 50% growth inhibition (IC_50_) in A2058 cells was 150 µM; therefore, this concentration was used in subsequent experiments. Clerosterol (150 µM) inhibited cell growth in a time-dependent manner (Figure 1b). Also, the cell viability of clerosterol at 150 µM was compared with 7β-hydroxycholesterol, a well-known cholesterol derivative with anticancer effect [15,16] in normal human keratinocyte HaCaT and the human melanoma cell line A2058. As shown in Figure 1c, cell viability of clerosterol and 7β-hydroxycholesterol at 150 µM was shown at 73% and 62% in HaCaT cells, respectively, and 49% and 60% in A2058 cells, respectively. These data demonstrated that clerosterol is more sensitive in cancer cells than normal cells. However, the cell viability of 7β-hydroxycholesterol was not shown.

### 2.2. Clerosterol Induces Apoptotic Cell Death in A2058 Cells

**Figure 2:**
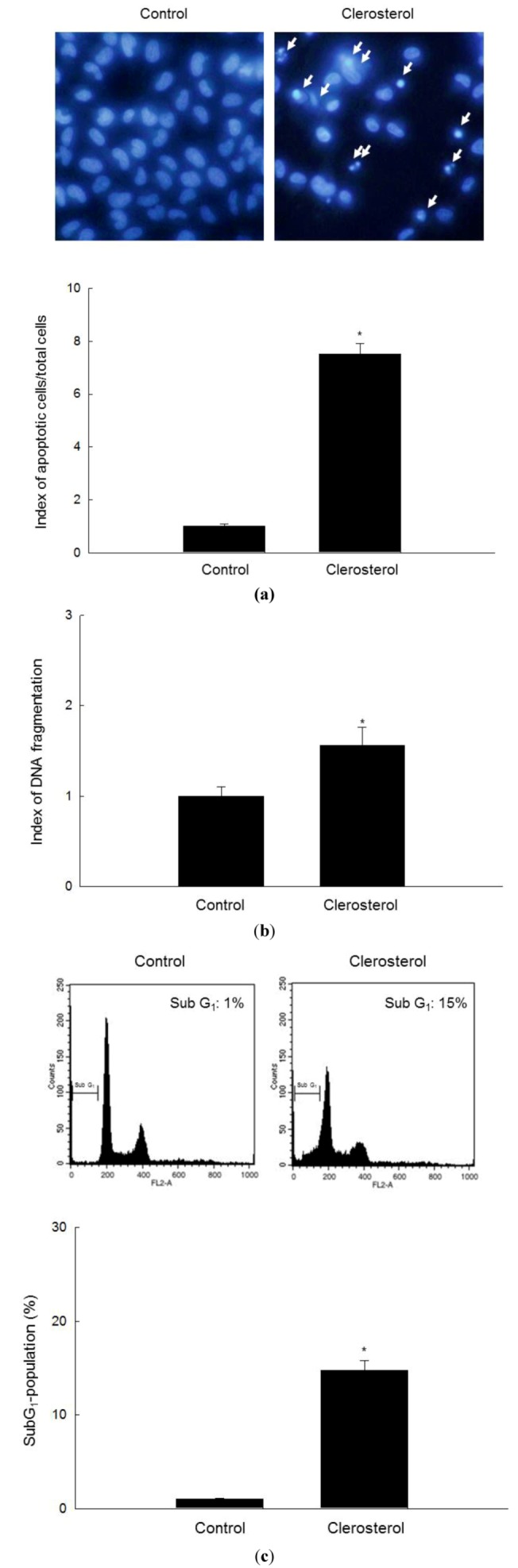
Induction of apoptosis by clerosterol. (**a**) The formation of apoptotic bodies (arrows) in Hoechst 33342-stained cells was observed by fluorescence microscopy and quantified. * Significantly different from control cells (*p* < 0.05). (**b**) DNA fragmentation was measured by ELISA. * Significantly different from control-treated cells (*p* < 0.05). (**c**) Sub-G_1_ cells were detected by flow cytometry after propidium iodide staining. * Significantly different from control cells (*p* < 0.05).

To determine whether the cytotoxicity of clerosterol is associated with the induction of apoptosis, the cell nuclei were stained with Hoechst 33342 and visualized by microscopy. Although control nuclei remained intact, significant nuclear fragmentation, which is characteristic of apoptosis, was observed in clerosterol-treated cells (Figure 2a). Moreover, the level of cytoplasmic histone-associated DNA fragmentation and the proportion of sub-G_1_-hypodiploid cells were higher in clerosterol-treated cells than in control cells (Figure 2b,c). 

### 2.3. Clerosterol-Induced Apoptosis Occurs through a Mitochondrial Pathway

**Figure 3:**
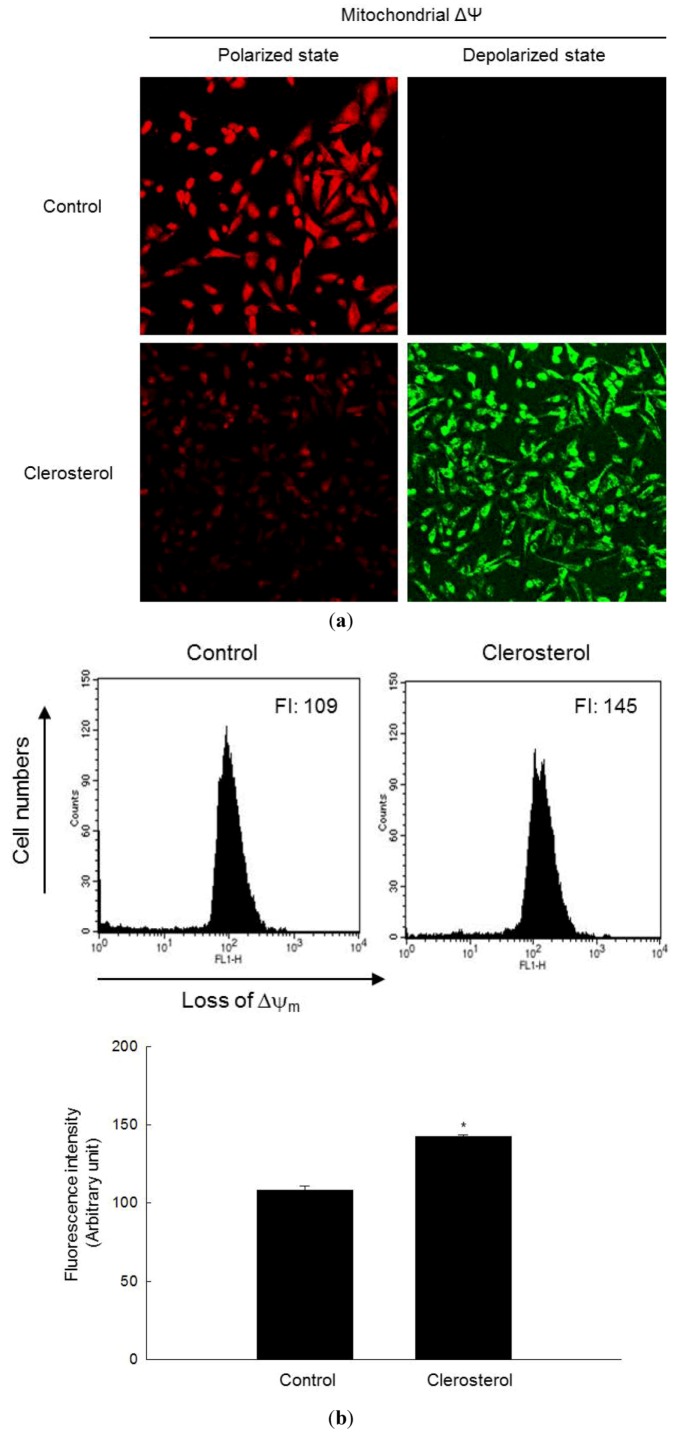
Loss of Δψm and induction of apoptosis by clerosterol. The Δψm was analyzed by confocal microscopy in cells stained with the dye JC-1 (**a**) and by flow cytometry (**b**). * Significantly different from control cells (*p* < 0.05).

The apoptotic pathway is associated with alterations in the mitochondrial membrane potential (Δψm), which led to mitochondrial membrane permeabilization, the release of cytochrome c and caspase activation [17]. The loss of Δψm in clerosterol-treated cells was confirmed by an increase in fluorescence intensity (FL-1) in cells labeled with the dye JC-1 (Figure 3a). The mitochondria in control cells showed strong red fluorescence, whereas those in clerosterol-treated cells showed decreased red fluorescence and increased green fluorescence, indicating the disruption of Δψm (Figure 3a). Flow cytometric analysis confirmed that clerosterol treatment caused a loss of Δψm, as evidenced by the higher intensity of JC-1 fluorescence in clerosterol-treated cells relative to that in untreated controls (Figure 3b).

### 2.4. Clerosterol Induces Apoptosis via Caspase Activation

**Figure 4:**
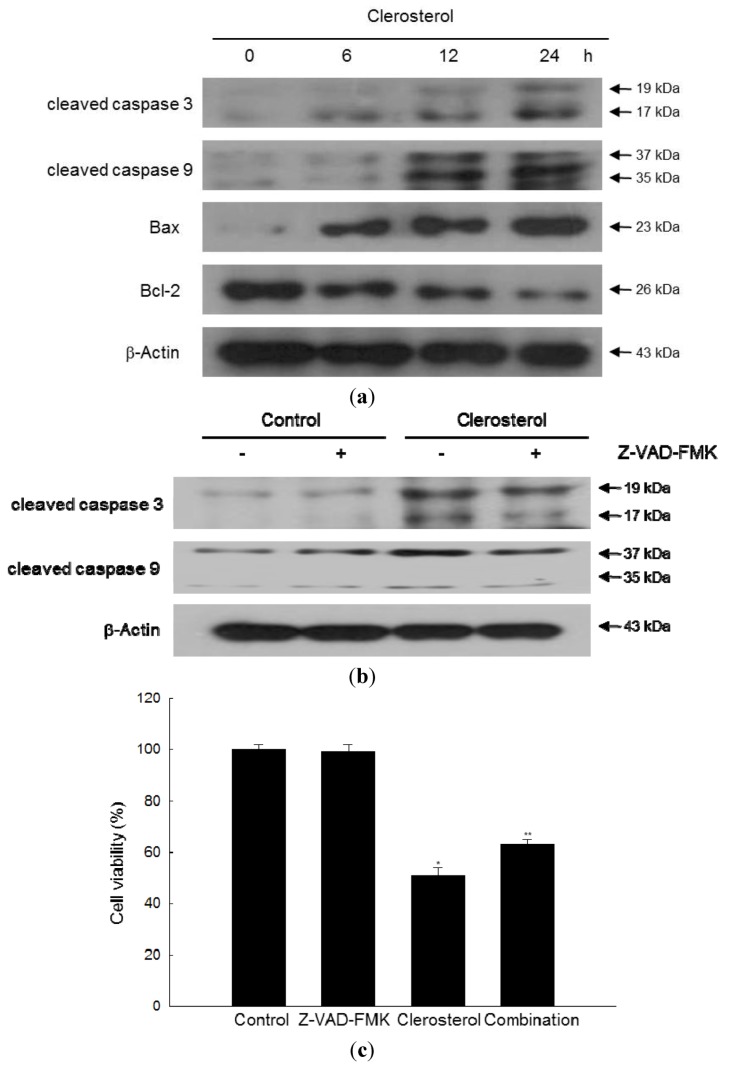
Involvement of caspases in clerosterol-induced apoptosis. (**a**) The clerosterol-treated cells were separated by electrophoresis, and cleaved caspase 3, cleaved caspase 9, Bax and Bcl-2 were detected by Western blotting with the corresponding antibodies. (**b**) The clerosterol-treated cells, Z-VAD-FMK-treated cells, Z-VAD-FMK and clerosterol-treated cells were separated by electrophoresis, and cleaved caspases were detected by Western blotting with the corresponding antibodies. (**c**) The cell viability of clerosterol-treated cells, Z-VAD-FMK-treated cells and clerosterol and Z-VAD-FMK-treated cells were detected by using MTT at 24 h.

To determine the potential involvement of caspases in clerosterol-induced apoptosis, we examined the effect of clerosterol on the expression or activation of key regulatory proteins associated with the caspases pathway. As shown in Figure 4a, clerosterol significantly increased the levels of the cleaved forms of caspases 3 and 9 in a time-dependent manner. Clerosterol treatment suppressed the expression of the anti-apoptotic protein Bcl-2 in A2058 cells and induced the expression of the pro-apoptotic protein, Bax. However, pan caspase inhibitor Z-VAD-FMK attenuated the cleaved forms of caspase 3 and 9, increased by clerosterol (Figure 4b) and led to inhibition of cell death induced by clerosterol (Figure 4c).

## 3. Discussion

Melanoma is a malignant tumor that originates in melanocytes. Although the incidence of melanoma is lower than that of other skin cancers, such as basal cell cancer and squamous cell cancer, it is more invasive and lethal than other skin cancers [18].

Apoptosis is a cell death process characterized by distinct morphological features and biochemical processes. The two major pathways of apoptosis, the extrinsic or death receptor pathway and the intrinsic or mitochondrial pathway, converge on the activation of the effector molecule, caspase 3 [19,20]. The extrinsic pathway is triggered by activation of death receptors at the plasma membrane, leading to the activation of caspase 8. The mitochondrial pathway is controlled by various pro- and anti-apoptotic proteins, such as the Bcl-2 family of proteins, which regulate the permeability of the mitochondrial membrane [21,22]. In addition, Bcl-2 directly inhibits members of the caspases family, including caspase 3 and caspase 9 [23,24]. On the other hand, the pro-apoptotic protein, Bax, promotes the loss of mitochondrial membrane integrity. Bax was identified as a Bcl-2-interacting protein, which opposes the action of Bcl-2 and promotes apoptotic cell death [25,26]. In a previous study, about three hundred species of marine algae from the coast of Japan were tested for *in vitro* antitumor activity, and four species of green algae showed strong cytotoxicity against the murine lymphoid leukemia L1210 cell line and low cytotoxicity to normal cells [27]. In another study, a glycoprotein derived from *Chlorella vulgaris* showed immunoreactive antitumor activity [28]. The mechanisms underlying *Chlorella vulgaris*-induced apoptosis were investigated in another study, which examined the expression and activity of the tumor suppressor protein, p53 and a host of pro- and anti-apoptotic proteins, such as Bcl-2, Bax and caspases 3 and 8 [29]. In animal studies, *Chlorella vulgaris* had anti-atherogenic, anti-cholesterolemic, anti-inflammatory and antitumor effects [30,31,32]. Also, an aqueous extract from the sea staghorn (*Codium fragile*) suppressed the growth of colon carcinoma cells by promoting apoptosis and downregulating the expression of the anti-apoptotic protein, Bcl-xL, leading to the activation of caspases 3 and 7 [33].

In the present study, cells treated with clerosterol showed chromatin condensation and DNA fragmentation, which are typical morphologic changes associated with apoptosis. Consistent with this observation, clerosterol treatment caused an increase in the hypodiploid cell population. The apoptosis-inducing effect of clerosterol was mediated via modulation of Bcl-2 and Bax expression, resulting in mitochondria-mediated apoptosis, and via an increase in the levels of cleaved caspases 3 and 9. 

## 4. Experimental Section

### 4.1. Reagents

7β-Hydroxycholesterol, [3-(4,5-dimethylthiazol-2-yl)-2,5-diphenyltetrazolium] bromide (MTT), Hoechst 33342 and propidium iodide were purchased from Sigma Chemical Co. (St. Louis, MO, USA) and 5,5′,6,6′-tetrachloro-1,1′,3,3′-tetraethylbenzimidazolylcarbocyanine chloride (JC-1) was purchased from Invitrogen (Carlsbad, CA, USA). Antibodies against caspase 9, caspase 3, Bcl-2 and Bax were purchased from Santa Cruz Biotechnology (Santa Cruz, CA, USA). Z-VAD-FMK, a pan-caspase inhibitor, was purchased from Tocris Bioscience (Minneapolis, MN, USA).

### 4.2. Cell Culture

The human melanoma cell line A2058 was purchased from Professor Hoi Young Lee (Konyang University, Nonsan, Korea). The Human keratinocyte HaCaT was obtained from the Amore Pacific Company (Gyeonggi-do, Korea). These cells were maintained at 37 °C in an incubator with a humidified atmosphere of 5% CO_2_ and cultured in DMEM containing 10% heat-inactivated fetal calf serum, streptomycin (100 μg/mL) and penicillin (100 units/mL).

### 4.3. Cell Viability Assay

The effect of clerosterol on cell viability was determined by the MTT assay, which is based on the reduction of a tetrazolium salt by mitochondrial dehydrogenase in viable cells [34]. Cells were seeded in 96-well plates at a density of 1 × 10^5^ cells/well and treated with clerosterol at a final concentration of 20, 60, 100, 150 or 200 μM. After 24 h, 50 μL of MTT stock solution (2 mg/mL) was added to each well to reach a total reaction volume of 250 μL, and the plates were incubated for an additional 4 h. The supernatants were aspirated, and the resulting formazan crystals were dissolved in 150 μL DMSO. The absorbance at 540 nm was measured using a scanning multi-well spectrophotometer. 

### 4.4. Nuclear Staining with Hoechst 33342

Cells were treated with clerosterol (150 μM) and incubated at 37 °C for 24 h. Hoechst 33342 (10 mg/mL stock; 1.5 μL), a DNA-specific fluorescent dye, was added to each well, and the cells were incubated for 10 min at 37 °C. The stained cells were visualized under a fluorescence microscope equipped with a CoolSNAP-Pro color digital camera (Media Cybernetics, Rockville, MD, USA). The degree of nuclear condensation was evaluated, and the number of apoptotic cells was quantified. The apoptotic index was calculated as (apoptotic cells in treated group/total cells in treated group)/(apoptotic cells in control group/total cells in control group).

### 4.5. DNA Fragmentation

Cells were treated with clerosterol (150 μM) and incubated at 37 °C for 24 h. Cellular DNA fragmentation was measured using a kit for quantifying cytoplasmic histone-associated DNA fragmentation (Roche Diagnostics, Indianapolis, IN, USA), according to the manufacturer’s protocol. 

### 4.6. Detection of Sub-G_1_ Hypodiploid Cells

The number of apoptotic sub-G_1_ hypodiploid cells was determined by flow cytometry [35]. Cells were treated with clerosterol (150 μM) for 24 h, harvested, washed twice with phosphate-buffered saline (PBS) and fixed in 70% ethanol for 30 min at 4 °C. After 30 min of incubation in the dark in a solution containing 50 mg/mL propidium iodide and 50 μg/mL RNase A, the cells were analyzed using a FACS Calibur flow cytometer (Becton Dickinson, Mountain View, CA, USA). The number of sub-G_1_ hypodiploid cells was determined based on histograms generated by the Cell Quest and Mod-Fit computer programs.

### 4.7. Analysis of Mitochondrial Membrane Potential (Δψm)

Cells were treated with clerosterol (150 μM) and incubated at 37 °C for 24 h. The cells were then stained with JC-1 (10 μg/mL) and analyzed by flow cytometry [36]. In addition, the JC-1-stained cells were mounted in mounting medium (DAKO, Carpinteria, CA, USA) and visualized under a confocal microscope. Image analysis was performed using the laser scanning microscope 5 PASCAL program (Carl Zeiss, Jena, Germany).

### 4.8. Western Blot Analysis

Harvested cells were washed in PBS, lysed in lysis buffer [120 mM NaCl, 40 mM Tris (pH 8), 0.1% NP 40] and centrifuged at 13,000× *g* for 15 min. Aliquots of the lysates (50 μg of protein) were boiled at 95 °C for 5 min and electrophoresed on SDS-polyacrylamide gels. The proteins in the gels were transferred to nitrocellulose membranes and probed with primary antibodies to Bax, Bcl-2, caspase-9 and caspase-3, followed by secondary immunoglobulin-G-horseradish-peroxidase conjugates. Reacting bands were visualized by enhanced chemiluminescence using a Western blotting detection kit (Amersham, Buckinghamshire, UK) coupled with a luminescent image analyzer. 

### 4.9. Statistical Analysis

All measurements were performed in triplicate and all values are expressed as the mean ± the standard error. The results were subjected to an analysis of variance (ANOVA), followed by Tukey’s test to analyze differences between conditions. In each case, a *p* value of <0.05 was considered statistically significant.

## 5. Conclusions

In conclusion, clerosterol isolated from the marine alga *Codium fragile* induces mitochondria-mediated apoptosis in A2058 human melanoma cells.
